# Prueba de cabecera para la valoración de la troponina de alta sensibilidad. Experiencia en el laboratorio clínico de un hospital terciario

**DOI:** 10.1515/almed-2024-0151

**Published:** 2024-11-18

**Authors:** Sarra El Amrani, Bastien Tossens, Louisa Van Belle, Judit Gonda, Sherine Midoun, Christophe Beauloye, Damien Gruson

**Affiliations:** Facultad de Farmacia UCLouvain, Bruselas, Bélgica; Servicio de Análisis Clínicos, Cínicas Universitarias St-Lux, Bruselas, Bélgica y UCLouvain, Bruselas, Bélgica; North-Pest Central Hospital – Hospital Militar, Budapest, Hungría; Unidad de Cardiología, Cínicas Universitarias Saint-Luc, Universidad Católica de Louvain, Bruselas, Bélgica; Centro de Investigación en Enfermedades Cardiovasculares, Instituto de Estudios Experimentales y Clínicos, Universidad Católica de Louvain, Bruselas, Bélgica; Centro de Investigación en Endocrinología, Diabetes y Nutrición, Instituto de Estudios Experimentales y Clínicos, Cínicas Universitarias Saint-Luc y UCLouvain, Bruselas, Bélgica; Department of Clinical Biochemistry, Cínicas UniversitariasSt-Luc et Université Catholique de Louvain, 10 Avenue Hippocrate, Brussels, 1200, Belgium

**Keywords:** Infarto de miocardio, troponina, biomarcador, point of care, eficiencia, coste

Estimado Editor,

Nos dirigimos a ustedes para compartir los conocimientos y experiencia adquirida en el empleo de pruebas de cabecera (POCT, *point-of-care testing*) para la determinación de la troponina cardíaca I de alta sensibilidad (HsTnI) en el centro Cínicas UniversitariasSaint-Luc (CUSL) de Bruselas (Bélgica). Este sistema ofrece un análisis rápido HsTnI, permitiendo obtener los resultados en ocho minutos, lo que puede mejorar significativamente la toma de decisiones clínicas, especialmente en el contexto del síndrome coronario agudo (SCA), donde un diagnóstico e intervención a tiempo resultan cruciales. La determinación de la troponina, junto con la evaluación de la historia clínica, síntomas, signos vitales y otros hallazgos del examen físico, resulta esencial en el diagnóstico del SCA [1]. La última guía de la Sociedad Europea de Cardiología recomienda taxativamente determinar la troponina cardíaca mediante valoración de alta sensibilidad en los intervalos 0 h/1 h o 0 h/2 h, así como disponer de los resultados del hemograma en menos de 60 minutos tras la extracción [1]. Las pruebas de cabecera (POCT, por sus siglas en inglés) suponen una alternativa a las pruebas tradicionales realizadas en el laboratorio clínico central, lo que permite realizar la prueba junto al paciente, reduciendo así el tiempo de respuesta [2, 3]. La nueva generación de pruebas POCT para la valoración de la troponina de alta sensibilidad ofrece la oportunidad de acelerar el proceso de triaje de los pacientes. El sistema Atellica VTLi^®^ es un dispositivo POCT con capacidad para analizar muestras de sangre total o plasma, habiendo mostrado un buen rendimiento clínico [4, 5] y analítico [6].

El objetivo del presente estudio es evaluar la manejabilidad del sistema Atellica VTLi^®^ POCT, para lo cual se emplearon muestras de plasma. Realizamos una encuesta a ocho profesionales sanitarios con distinta capacitación. El cuestionario se diseñó aplicando la Evaluación Escandinava de Equipos de Laboratorio para Cuidados Primarios (SKUP) [7]. Se pidió a los participantes que asignaran una valoración (satisfactorio, aceptable, insatisfactorio) a cada una de las cuestiones (11 en total). Huelga decir que el objetivo de manejabilidad de todo sistema es lograr una puntuación total de ‘satisfactorio’. Tal como muestran los resultados de la Tabla 1, las respuestas fueron abrumadoramente positivas, ya que los usuarios indicaron un elevado nivel de satisfacción con respecto al peso del dispositivo, la descripción de la prueba y el procedimiento de extracción de la muestra. Cabe señalar que los participantes indicaron que no era necesario recibir formación específica para su uso, ya que el método para realizar la prueba es sencillo e intuitivo. Este aspecto de facilidad de uso resulta de especial relevancia en el contexto de las urgencias, donde la manejabilidad puede influir notablemente en la eficiencia de los flujos de trabajo. El tamaño del dispositivo, así como el material requerido para la realización de la prueba recibieron la calificación de aceptables, lo que respalda su utilidad en diversos contextos clínicos. La calificación de “aceptable” subraya el hecho de que, si bien el sistema POCT resulta muy útil para el diagnóstico urgente, ampliar sus aplicaciones puede requerir una mayor capacitación, modificar los protocolos clínicos, o llevar a cabo su validación, con el fin de optimizar el empleo de estos sistemas en la predicción de riesgos y el seguimiento continuo del paciente. Estas valoraciones matizadas enfatizan la importancia de contar con estrategias de implementación adaptadas que aborden necesidades y escenarios clínicos concretos.

**Tabla 1:** Encuesta.

Criterios	Satisfactorio (S)	Aceptable (I)	Insatisfactorio (I)	Resultados
Peso del dispositivo	<5 kg	>5 kg × <10 kg	>10 kg	SSSSSSSI
Tamaño del dispositivo	<5 cm × 5 cm	>5 cm × 5 cm × <10 cm × 10 cm	>30 cm × 30 cm	SSSIIIII
Equipamiento adicional necesario para realizar el análisis (dispositivo, cartuchos, consumibles)	No	1–2	>3	SSSSSSII
Descripción del proceso de extracción de la muestra	SÍ	Incompleto	No	SSSSSSSS
Descripción del proceso de análisis	SÍ	Incompleto	No	SSSSSSSS
Descripción de la presentación de los resultados (y las unidades empleadas son las mismas que las empleadas en los análisis ordinarios)	SÍ	Incompleto	No	SSSSSSII
Función y uso de la prueba (en qué casos se puede emplear: diagnóstico / predicción de riesgos / monitorización)	SÍ	Incompleto	No	SSSSIIII
Presencia de las instrucciones necesarias para que el usuario realice la prueba correctamente	SÍ	Incompleto	No	SSSSSSSS
Formación necesaria para obtener las muestras	No	Incompleto	SÍ	SSSSSSSS
Formación requerida para utilizar el dispositivo	No	Formación básica	Formación especial	SSSSSSII
Es necesario recibir formación para la calibración y el control de calidad del dispositivo (disponibilidad de instrucciones para llevarlas a cabo)	SÍ	Incompleto	No	SSSIIIIU

**Figura 1:**
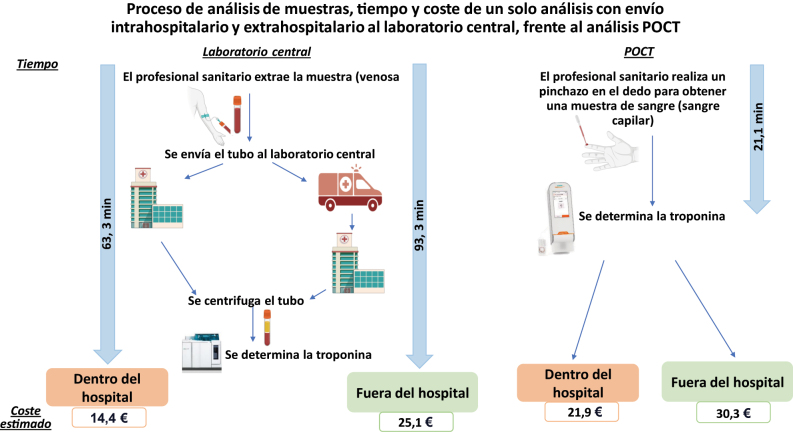
Proceso de análisis de muestras mediante POCT frente al laboratorio central de Saint-Luc. Tiempo y coste de una sola prueba en el envío intra y extrahospitalario and cost al laboraotrio central, comparado con el dispositivo VTLi.

Otro de los objetivos del estudio era realizar una comparación del itinerario POCT con el itinerario del laboratorio central, en cuanto a costes y tiempos de respuesta (TDR). Para tal fin, se realizó un registro y análisis meticuloso de los pasos realizados desde la admisión del paciente hasta la recepción de los resultados. Así, se registraron los tiempos de respuesta de los dos métodos, esto es, mediante el envío al laboratorio central y mediante la realización de la prueba POCT *in situ*. El tiempo de respuesta se definió como el tiempo transcurrido desde la extracción de la muestra hasta la recepción del resultado. Calculamos el coste de realizar la prueba POCT con el sistema Atellica VTLi^®^, así como el coste de realizar el análisis en el laboratorio central, realizando un análisis completo de los costes asociados a los dos itinerarios. Se incluyeron tanto los costes directos como los indirectos asociados al proceso de análisis. Tanto para la prueba POCT como para la prueba en el laboratorio central, se incluyeron consumibles comunes, tales como hisopos con alcohol, guantes, vendajes adhesivos, jeringas, agujas y tubos, con consumibles específicos para POCT, que incluyen lancetas y cartuchos de prueba. Entre los costes de los equipos e instrumentación para la prueba POCT, se incluyeron el dispositivo Atellica VTLi^®^, el software del ordenador, los reactivos de control de calidad, un refrigerador y el mantenimiento, mientras que los costes del laboratorio central incluyeron instrumentos, reactivos de control de calidad y mantenimiento. Los costes de personal se calcularon en función del tiempo dedicado por los profesionales sanitarios a la extracción de la muestra, su análisis y el procesamiento del resultado, aplicando honorarios por hora. Los costes de transporte de las muestras al laboratorio central incluyeron el transporte intrahospitalario mediante tubo neumático y el transporte terrestre desde los centros de atención primaria, que desaparecen en el itinerario POCT. Así mismo, se tuvieron en cuenta los gastos generales y de infraestructura relacionados con el espacio del laboratorio y los suministros. El tiempo de respuesta fue considerablemente menor en el itinerario POCT. Así, la mediana de TDR de la prueba POCT fue de 21,1 minutos, considerablemente más rápido que el análisis en el laboratorio central, con un tiempo de respuesta que podía alcanzar los 93,3 minutos. A pesar de mostrar un TDR más rápido, la prueba POCT supuso unos costes directos levemente superiores. El coste de la prueba POCT en el hospital fue de 21,9 €, comparado con los 14,4 € del análisis en el laboratorio central. Del mismo modo, el coste de la prueba POCT fuera del hospital fue de 30,3 €, frente a los 25,1 € del análisis en el laboratorio central. En la Figura 1 se muestra un resumen de los resultados.

Aunque la prueba POCT tiene unos costes directos mayores, el beneficio económico global justificaría la inversión. El rápido tiempo de respuesta ofrecido por el sistema POCT podría suponer un ahorro en costes indirectos. Por ejemplo, un diagnóstico y tratamiento más rápidos podrían reducir la duración de la estancia hospitalaria, optimizar el consumo de recursos sanitarios y mejorar el flujo de pacientes. Estudios previos revelan que los algoritmos rápidos basados en los niveles de troponina presentan un mayor costo efectividad que el manejo estándar, logrando reducciones significativas en los costes y en la duración de la estancia hospitalaria [8]. Así mismo, no hay que olvidar el menor impacto medioambiental de la prueba POCT. Nuestra vía ambulatoria actual implica el transporte en coche de las muestras hasta el laboratorio central. La prueba POCT, por el contrario, proporciona los resultados *in situ*, eliminando el transporte terrestre de las muestras, y reduciendo así su huella medioambiental. Este aspecto contribuye a unas prácticas sanitarias más sostenibles, en consonancia con los objetivos más amplios de responsabilidad medioambiental.

En conclusión, nuestra experiencia preliminar con el sistema POCT Atellica VTLi^®^ para la determinación HsTnI en un hospital terciario demuestra un gran potencial a la hora de mejorar la toma de decisiones clínicas, gracias a su menor tiempo de respuesta. En el manejo del SCA, donde “el tiempo es músculo”, mejorar la capacidad de obtener resultados HsTnI y actuar en consecuencia podría influir significativamente en los resultados clínicos del paciente. El sencillo diseño del sistema y las experiencias positivas de los usuarios respaldan la utilidad de este sistema en diversos contextos clínicos. Si bien la prueba POCT implica mayores costes directos, sus beneficios económicos generales, incluyendo un triaje más rápido, así como la optimización en el consumo de recursos, respaldan su implementación. Es necesario realizar estudios más amplios para evaluar con mayor precisión el impacto económico de la implementación de este sistema y validar los resultados del presente estudio.
